# Adaptive HIV-1 evolutionary trajectories are constrained by protein stability

**DOI:** 10.1093/ve/vex019

**Published:** 2017-08-01

**Authors:** Abayomi S. Olabode, Shaun M. Kandathil, Simon C. Lovell, David L. Robertson

**Affiliations:** 1 Evolution & Genomic Sciences, School of Biological Sciences, University of Manchester, Oxford Road, Manchester, UK; 2 Francis Crick Institute & Dept. of Computer Science, University College London, London, UK; 3 MRC-University of Glasgow Centre for Virus Research, Garscube Campus, Glasgow, UK

**Keywords:** HIV-1, evolution, drug resistance, protein structure, protein stability

## Abstract

Despite the use of combination antiretroviral drugs for the treatment of HIV-1 infection, the emergence of drug resistance remains a problem. Resistance may be conferred either by a single mutation or a concerted set of mutations. The involvement of multiple mutations can arise due to interactions between sites in the amino acid sequence as a consequence of the need to maintain protein structure. To better understand the nature of such epistatic interactions, we reconstructed the ancestral sequences of HIV-1’s Pol protein, and traced the evolutionary trajectories leading to mutations associated with drug resistance. Using contemporary and ancestral sequences we modelled the effects of mutations (i.e. amino acid replacements) on protein structure to understand the functional effects of residue changes. Although the majority of resistance-associated sequences tend to destabilise the protein structure, we find there is a general tendency for protein stability to decrease across HIV-1’s evolutionary history. That a similar pattern is observed in the non-drug resistance lineages indicates that non-resistant mutations, for example, associated with escape from the immune response, also impacts on protein stability. Maintenance of optimal protein structure therefore represents a major constraining factor to the evolution of HIV-1.

## 1. Introduction

HIV-1 is the etiologic agent for Acquired Immunodeficiency Syndrome (AIDS) (Barre-Sinoussi et al. 1983; Gallo et al. 1983) and is estimated to have been in the human population since at least the 1920s (Faria et al. 2014). Approximately 35 million people are currently infected with HIV-1, and since its discovery over 30 years ago, there have been over 39 million deaths (Gulland 2014). AIDS is characterized by progressive immune system failure followed by loss of immune function, and subsequent development of cancers and opportunistic infections that lead to death of infected individuals (Gallo and Montagnier 2003). Treating infected individuals effectively has been difficult due to the presence of latent virus and reservoirs necessitating continued therapy (Lorenzo-Redondo et al. 2016), the evolution of drug resistant HIV-1 strains and failure of vaccine initiatives (Greene 2007).

Highly active antiretroviral therapy (HAART) delays the progression to disease, prolonging the lives of infected individuals, and reducing the transmission of the virus (Cooper and Merigan 1996; Rhee et al. 2003; Shafer 2006; Falco et al. 2008). The use of antiretroviral drugs has been associated with the accumulation of missense mutations causing amino acid replacements that lead to the emergence of drug resistance. These changes are examples of adaptive viral evolution: typically selection pressure arising from the host immune system or the presence of antiretroviral drugs (Pillay et al. 2000; Rambaut et al. 2004). Drugs fall into several classes: nucleoside reverse transcriptase inhibitors (NRTIs) and non-nucleoside reverse transcriptase inhibitors (NNRTIs), both targeting reverse transcriptase, other inhibitors targeting the protease or integrase (Furman and Barry 1988; Reeves and Piefier 2005; Evering and Markowitz 2008), or fusion and entry inhibitors that are designed to block cell entry (MacArthur and Novak 2008; Eggink et al. 2010). Unfortunately, resistance to all of these drug classes has been observed.

Resistant strains are usually fixed in the population as a result of competition between variants of HIV-1 in the context of drug selection (Ribeiro and Bonhoeffer 2000). Despite a high degree of sequence diversity, the virus is subject to constraints on its evolution. These constraints arise principally from the requirement of the viral proteins to produce stable, soluble structures that perform the necessary molecular functions for viral replication and persistence (Woo et al. 2010; Snoeck et al. 2011; Williams et al. 2011). Co-evolution within individual HIV-1 proteins has been identified as an important constraint in the evolution of immune escape (Dahirel et al. 2011). Similarly, HIV-1 drug resistance can be restricted by epistatic interactions (Bonhoeffer et al. 2004; Chen et al. 2004; Hinkley et al. 2011), which apparently arise predominantly from residues that are in close proximity in the protein structure (Hinkley et al. 2011).

The majority of mutations that can possibly occur in protein structure (for all species) have been shown to be destabilizing and, hence, deleterious with only about 5% having a stabilizing effect (ΔΔG < −1 kcal/mol) (Tokuriki et al. 2007). That the majority of possible changes result in non-functional proteins therefore poses a major constraint on possible future adaptations (Tokuriki and Tawfik 2009). As a consequence it is expected that protein stability will constrain possible change, with increased stability a pre-requisite for significant evolutionary change, that is, the acceptance of otherwise deleterious destabilizing amino acid replacements (Bloom et al. 2006; Tokuriki and Tawfik 2009). For example, a small number of protease residue replacements have been characterized experimentally (Chang and Torbett 2011), demonstrating the importance of ‘permissive’ changes for the evolution of otherwise deleterious replacements, and the importance of residue replacements away from the active site in altering binding affinity for protease inhibitors (Muzammil et al. 2003).

Here, we investigate the importance of this stability mechanism in the HIV-1 pandemic by analysing relatively large sets of viral sequences in the context of three-dimensional structures of Pol proteins and their evolutionary history. By reconstructing ancestral sequences and mapping amino acid replacements to the protein structures, we can characterize the likely effects of replacements on protein structure, stability and function. Although the majority of resistance-associated sequences destabilise the protein structure, this is the general trend for many residue changes. Specifically, comparing HIV-1’s more recent evolutionary history with inferred ancestral sequences, there has been a significant decrease in protein stability. We discuss the implications of these results for the fitness of circulating HIV-1.

## 2. Results

### 2.1 Phylogenetic analysis

**Figure 1.**
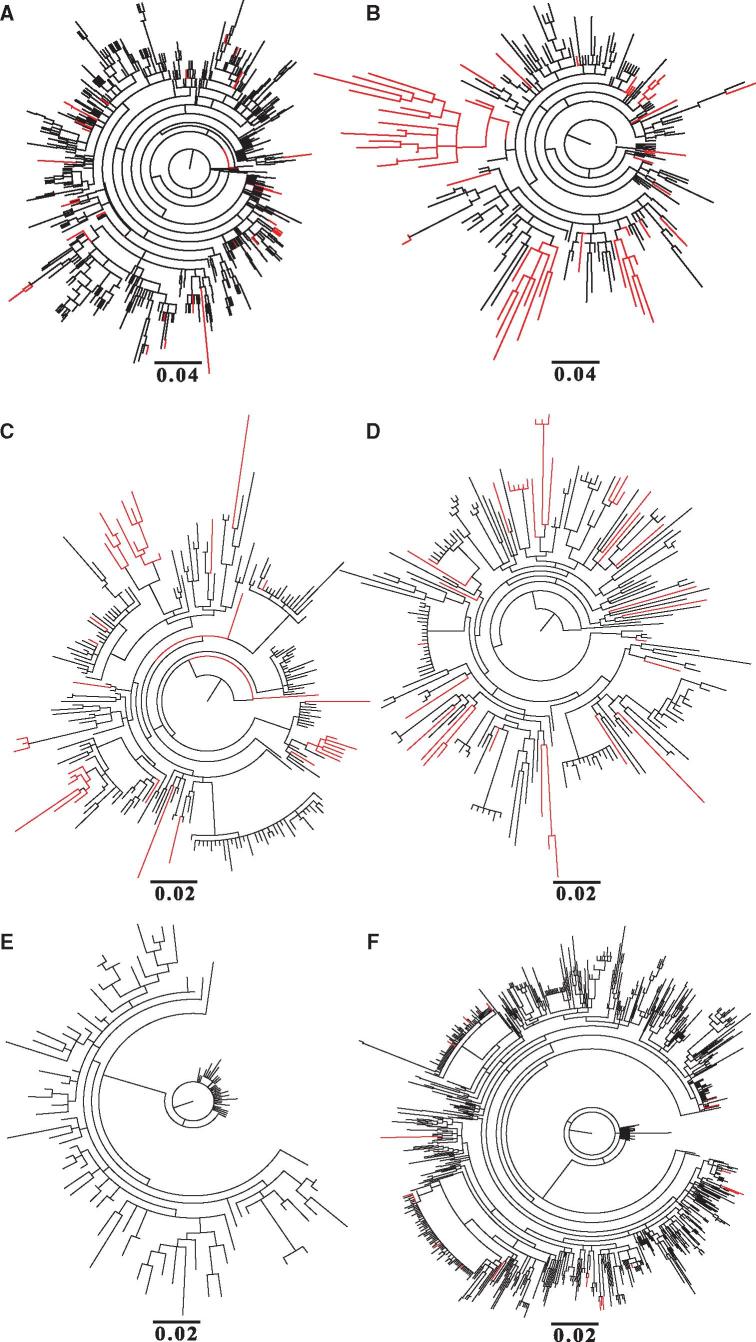
Phylogenetic trees showing the evolutionary history of HIV-1 subtype B sequences: pre-HAART/1996 protease sequences (A), post-HAART/1996 onwards protease sequences (B), pre-HAART/1996 reverse transcriptase sequences (C), post-HAART/1996 onwards reverse transcriptase sequences (D), pre-HAART/1996 integrase sequences (E), and post-HAART/1996 onwards integrase sequences (F). The branches coloured red represent those sequences obtained from the Los Alamos HIV-1 sequence database that have at least one mutation conferring resistance to protease inhibitors (A & B), NRTIs/NNRTIs (C & D) and integrase inhibitors (E & F), whereas those coloured black have no identified drug resistance mutations. The scale bar below each tree indicates the number of amino acid replacements per site.

To determine the effect of drug selection on the emergence of resistance, we aligned subtype B sequences from the Los Alamos National Laboratory database that were dated as either pre-1996 or 1996 onwards, as this is the year that HAART was widely introduced. We assume the sequences sampled post-1996 to be from patients receiving HAART, since it is likely that all patients who contributed samples for sequencing in the USA and Europe (where subtype B is mostly found) would be receiving drug treatment. Maximum likelihood trees for the protease protein (Fig. 1A) show that in the absence of drug treatment, sequences containing amino acid replacements known to confer drug resistance, as expected, are sparse and scattered throughout the phylogenetic tree. A similar pattern is observed for the reverse transcriptase proteins (Fig. 1C). No resistance conferring mutations are observed in the sequences from the pre-HAART era for the integrase protein (Fig. 1E). For the post-HAART data, we observed many more amino acid replacements conferring resistance to protease inhibitors, and these sequences were often clustered in the phylogenetic tree (Fig. 1B). Similar patterns were seen for the reverse transcriptase and integrase proteins (Fig. 1D and F). Comparable results were observed when the phylogenetic analysis was repeated with all sites in the protein sequence known to confer drug resistance removed, indicating that the patterns are not due to convergent evolution (Supplementary Fig. S1).

**Figure 2.**
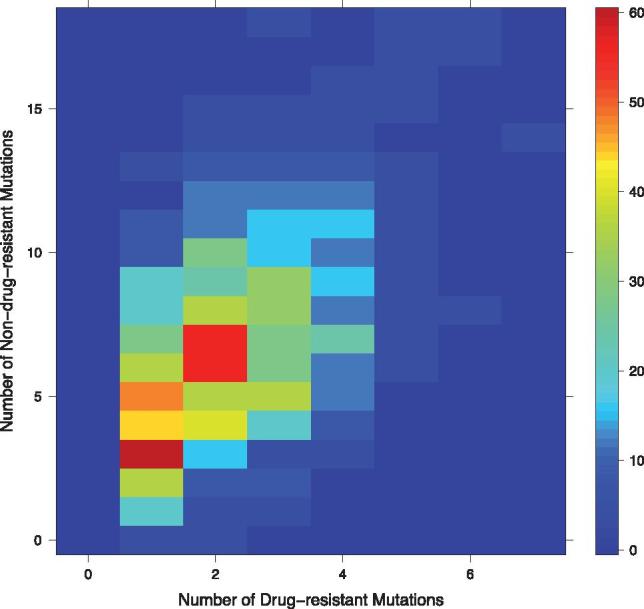
Heatmap plot showing the correlation between the frequency of drug resistance mutations (counts of amino acid replacements) in a sequence and the number of non-drug resistance mutations accumulated from 1996 onwards in protease sequences. The *y*-axis indicates the frequency of non-drug resistance mutations accumulated with respect to the number drug resistance mutations, while the *x*-axis shows the number drug resistance mutations. The colour of the square bins indicates the number of sequences in the data that have the frequency of non-drug resistance associated mutations.

Analysis of the sequence data sets showed that 85% of the sequences containing drug resistance-associated mutations are associated with up to three changes in protease (Fig. 2), and that there is a significant correlation between the acquisition of drug resistance associated mutation and the accumulation of associated non-drug resistance mutations (Pearson’s *r* = 0.5, *p* < 0.05). For example, a sequence having as many as five drug resistance mutations could be associated with as high as 14 non-resistant mutations (Fig. 2). This pattern suggests that drug resistance occurs randomly with more resistance conferring changes associated with more evolutionary change. Note that we are cautious about over-interpreting any branch length differences. The drug resistant viruses are presumably from uncontrolled infections so are more likely to have been transmitted, and also to have accumulated more variation (so exhibiting longer branches).

### 2.2 Structural analysis

In order to characterize the likely effects of variants on protein function and viral fitness we mapped protein sequence changes to the protein structure and characterized their impact. Using maximum likelihood methods, we reconstructed the likely ancestral sequences for each node of the phylogenetic trees for the data sampled from 1996 onwards. Using standard comparative modelling techniques (Sali and Blundell 1993), we built structural models of the inferred ancestral node sequences and traced the likely evolutionary trajectory for each external node containing at least one drug resistance-conferring amino acid replacement.

**Figure 3.**
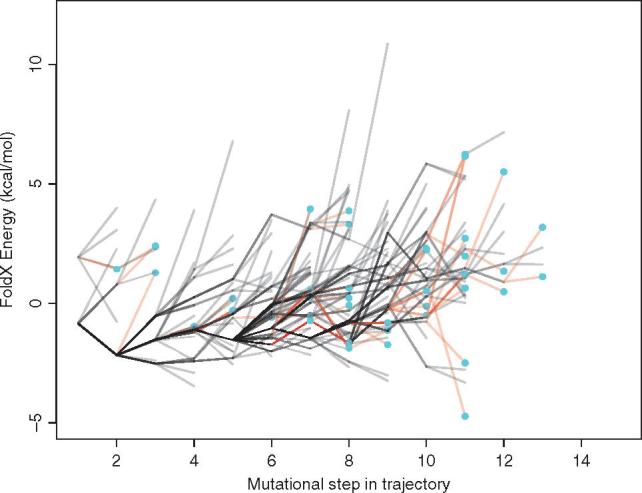
Energy landscape showing trajectory energies for HIV-1 protease protein for both resistant and non-resistant sequences. Each trajectory is plotted in grey, with frequently shared paths appearing to be darker. The red lines indicate the step in resistance trajectories just before a resistance mutation is acquired (indicated by the blue circles). The *y*-axis represents the predicted difference in energy between each mutant and the wild-type strain (ΔΔG). Each line on the *x*-axis represents a mutational step between a parent and child node starting from the first descendant of the most recent common ancestor. Note, the energy changes are cumulative.

**Figure 4.**
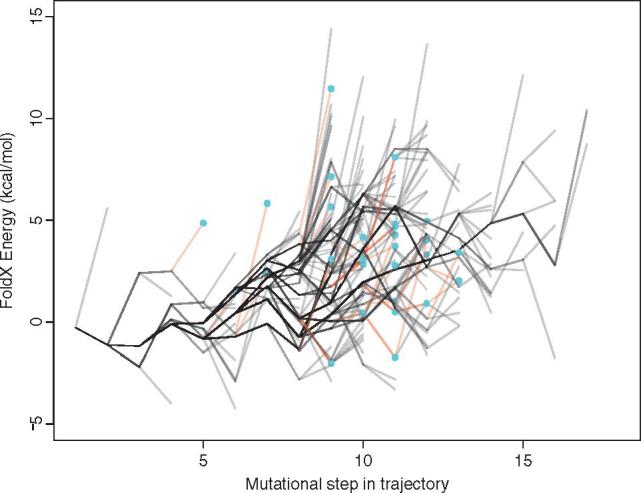
Energy landscape showing a summary of trajectory energies for HIV-1 reverse transcriptase protein for both resistant and non-resistant sequences. See Fig. 3 legend for details.

**Figure 5.**
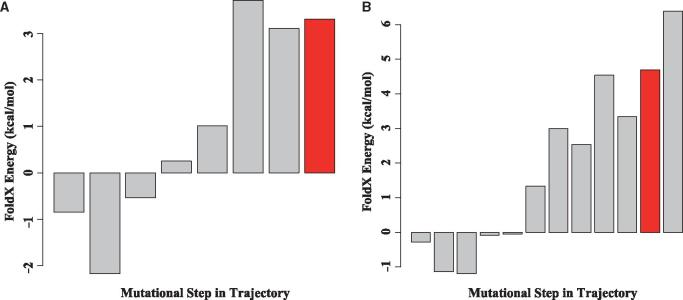
Example landscape of the energy changes for each internal node in a trajectory that leads to a resistance mutation for HIV-1’s protease protein (A) and reverse transcriptase protein (B). The *y*-axis represents the predicted difference in energy between each mutant and the wild-type strain (ΔΔG). Each bar on the *x*-axis represents a mutational step between a parent and child node starting from the first descendant of the most recent common ancestor. The red bar indicates the node (step) where the drug resistance conferring mutation occurred.

**Figure 6.**
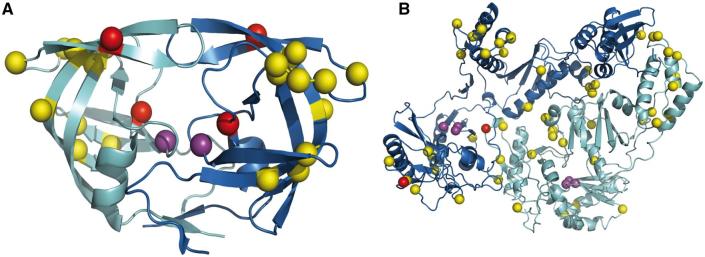
Structural context of the drug resistance mutations (amino acid replacements) occurring along an example trajectory in HIV-1’s protease protein, PDB code 1KZK (A) and reverse transcriptase protein, PDB code 1RTJ (B), see Fig. 5 for trajectories. The resistance mutations for the protease (M46I and I84V) and reverse transcriptase are coloured red. The catalytic aspartates are coloured purple. The various amino acid replacements occurring prior to the resistance mutation are shown in yellow.

The effect of amino acid replacements on protein stability was predicted for all ancestral sequences in each trajectory using FoldX (Guerois et al. 2002). We find that the sequences in the early parts of most trajectories are characterized by amino acid replacements that have a neutral or stabilizing impact on the protein structure (Figs 3 and 4). Examination of an example trajectory for each protein (Fig. 5) shows that the later part of each trajectory is categorized by a high frequency of destabilizing mutations, and many of these replacements are associated with drug resistance. We mapped the mutations occurring along all mutational steps occurring in these evolutionary trajectories to the HIV-1 protein structures. Interestingly, amino acid replacements tend not to be found in close proximity to the resistance mutations, but instead are spread throughout the protein structure (Fig. 6).

**Figure 7.**
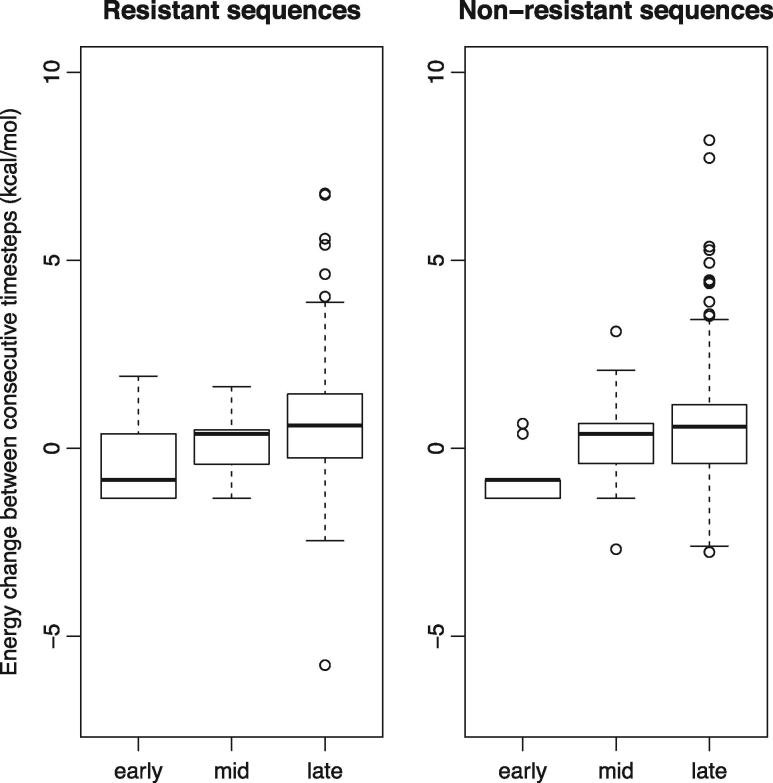
Box and whisker plots comparing the distributions of ΔΔG values in the protease protein for early, middle and late stages of the evolutionary trajectories for all extant sequences for resistant and non-resistant sequences.

**Figure 8.**
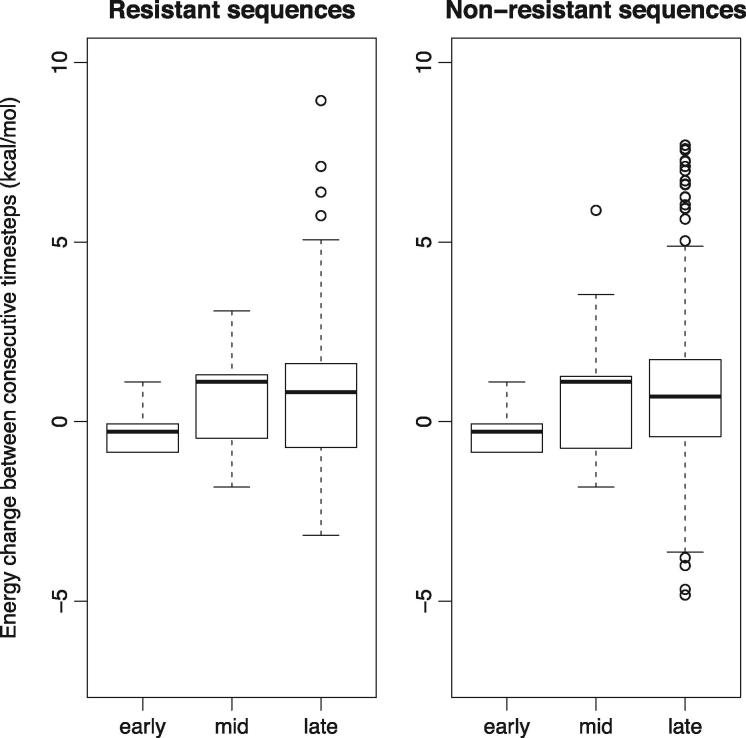
Box and whisker plots comparing the distributions of ΔΔG values in the reverse transcriptase protein for early, middle and late stages of the evolutionary trajectories for all extant sequences for resistant and non-resistant sequences.

In order to compare protein stability across the trajectories, each was split into early, middle and late parts. Comparing these parts for all trajectories, we find there is a general trend of decreasing protein stability (Figs 7 and 8). Surprisingly, the same trend is observed for the non-resistant lineages (Figs 7 and 8). Testing this, we find that there is a significant difference (Mann–Whitney Test, *p* < 0.05) between the ΔΔG values of the early versus middle and middle verse late parts of the trajectories in both the resistant and non-resistant sequences for the protease protein. The pattern is similar for reverse transcriptase for early verse middle parts of the trajectories in both the resistant and non-resistant sequences, and only significant for the middle versus late parts for the non-resistant sequences (*p* < 0.05).

These analyses were repeated with different random samples of trajectories (200 in total), and these showed similar trends. To compare the replicate trajectory sets (Supplementary Figs S4 and S5), we assessed the high-energy portions of the trajectories by considering the 75th percentile values of all cumulative energy values seen in each trajectory. For the trajectories leading to drug resistance in the protease protein, we find that the 75th percentile value does not exceed 4.56 kcal/mol across the five samples, and the average of the 75th percentile across the five samples was 3.47 kcal/mol. On the other hand, the resistance trajectories for reverse transcriptase had a 75th percentile value which does not exceed 8.67 kcal/mol, and the average of the 75th percentile across the five samples was 6.92 kcal/mol. The average of the 75th percentile across the five samples for the non-resistance trajectories encountered in the protease protein was 2.35 and 6.48 kcal/mol for reverse transcriptase.

Although these findings confirm the tendency for significant amino acid changes to destabilise the protein structure, they indicate that there’s a general decrease in the protein stability across HIV-1’s recent evolution. To check this pattern is not due to the resistant and non-resistant variants sharing ancestry, we repeated the analysis excluding the resistant sequences. Our results demonstrate that the trajectories were similarly characterized by early stabilizing mutations that precede destabilizing mutations (data not shown). This must be a reflection of the necessity for the virus to escape the immune response via residue replacements, and as such these non-resistant lineages cannot be considered as controls as they are also under-going adaptive evolution.

**Figure 9.**
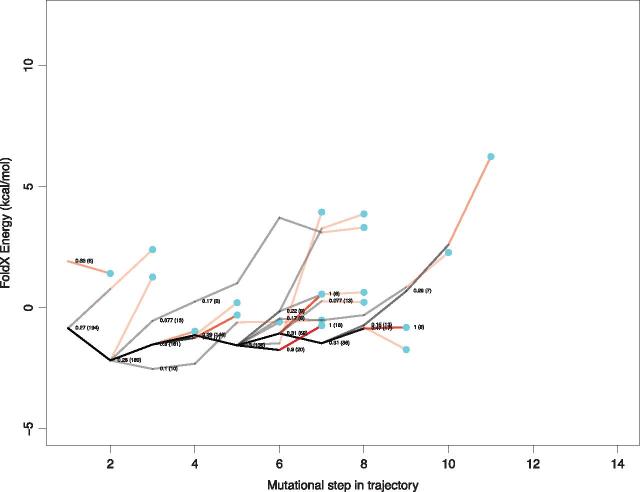
Energy landscape showing a summary of the cumulative energy changes for all trajectories including those leading to drug resistance in the HIV-1 protease protein. The *y*-axis represents the predicted difference in energy between each mutant and the wild-type strain (ΔΔG). Each line on the *x*-axis represents a mutational step between a parent and child node starting from the first descendant of the most recent common ancestor. Each trajectory is terminated at the first mutational step where a drug resistant mutation is detected. The red lines indicate the step in each trajectory just before a resistance mutation is acquired indicated by the blue circles. The probability of a sequence leading to a resistance-associated mutation is shown (total number of sequence passing through that node is in parentheses). The probability of acquisition of a resistance mutation is calculated by dividing the total number of pathways passing through a node that ultimately acquire a resistance mutation by the total number of pathways. For clarity values are omitted if fewer than five sequences pass through a node.

**Figure 10.**
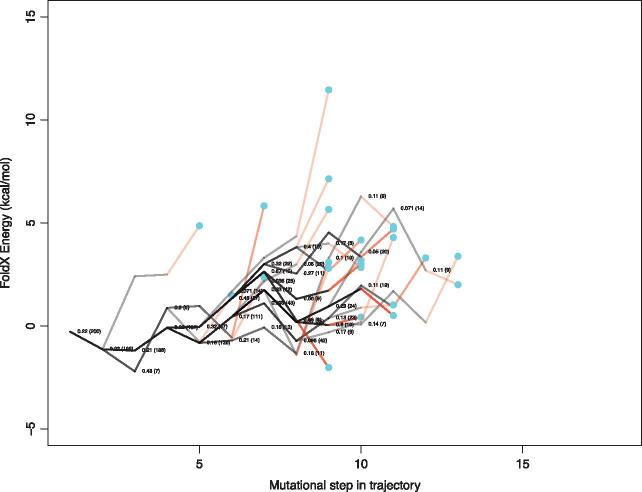
Energy landscape showing a summary of the cumulative energy changes for all trajectories including those leading to drug resistance in the HIV-1 reverse transcriptase protein. See Fig. 9 legend for details.

Finally, we estimated the probability of viral sequences following a pathway that will lead to the acquisition of drug resistance. We find that certain nodes in some trajectories lead to the development of resistance more frequently as indicated by higher probability scores (Figs 9 and 10). Sequences passing through the ancestral nodes that carry a particular set of mutations are therefore more likely to eventually acquire a drug resistance mutation.

**Figure 11.**
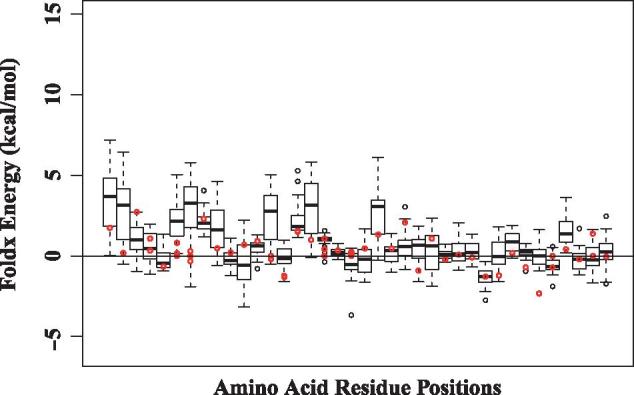
Analysis of mutations (amino acid replacements) in the context of the HIV-1 integrase protein structure. Change in energy (ΔΔG) for all amino acid replacements found in regions of known protein structure, as predicted using a statistical potential. Each boxplot represents a distribution of energy changes to all 19 other residue types at positions where a non-synonymous substitution has been observed. The ΔΔG of the observed substitution is indicated in red.

Because of the lack of a complete three-dimensional structure covering the whole length of the integrase protein, we investigated the degree to which amino acid replacements can be tolerated in those positions in the partial structure where mutations were observed. Using FoldX, we computed the energy changes associated with the observed amino acid replacements against a background distribution of energies of mutations to the 19 other possible amino acids. We found that the change in folding energies for the observed amino acid replacements were mostly stabilizing or neutral with a small number that were destabilizing. When compared to the background distributions of the change in folding energies, the predicted effect of the observed amino acid replacements were relatively small, meaning that the observed replacements are likely to have minimal impact on the stability of the HIV-1 integrase protein (Fig. 11). However, for the few cases where the observed amino acid replacement energy value was above the upper quartile of the energy distributions, we found that these replacements occurred later in the evolutionary trajectories after the enabling substitutions had occurred, confirming that stabilizing mutations usually precede destabilizing ones.

## 3. Discussion

Collectively, our results indicate that drug resistance is constrained by fitness effects arising from protein structure, in a similar way to protein stability constraints that need to be overcome for immune escape to occur (Boutwell et al. 2013). This is consistent with a subset of the resistance mutations that have been tested experimentally by Chang and Torbett (2011). Their analysis on both point and double mutants demonstrated that the accumulation of drug resistance associated mutations destabilises the HIV-1 protease protein. Chang and Torbett also showed that specific non-drug resistant accessory mutations, which are stabilizing, are acquired to stabilise the protein, supporting our modelling results. Over the course of our evolutionary trajectories, the total energy change (cumulative ΔΔG of replacements) tends to be mostly neutral or positive for the HIV-1 proteins, resulting in trajectories that are destabilizing overall.

Interestingly, similar trajectories are observed in the non-drug resistance lineages (Figs 3 and 4) indicating this type of evolution is a normal part of HIV-1 evolution due presumably to the need to accommodate otherwise deleterious/destabilizing mutations in the context of on-going immune escape (Boutwell et al. 2013). What is particularly interesting is this tendency for viruses to have accumulated destabilizing mutations in an infection will impact on their transmissibility such that transmitted variants will tend to be ‘purged’ of destabilizing mutations (Carlson et al. 2014). This would also explain why drug resistance variants are often not successful when transmitted or often revert when drug treatment is stopped (Devereux et al. 1999; Verhofstede et al. 1999).

Crucially, the consequences of a missense mutation must be assessed in the context in which it occurs. A mutation that is deleterious in one context may be beneficial in another and this will depend on the stability of the specific protein structure. Furthermore, mutations may individually lead to beneficial changes for one aspect of molecular function but may be deleterious with regard to another aspect. We have observed many amino acid replacements of this type, in that they have the potential to convey drug resistance but at the same time reduce protein stability. Indeed most viral populations revert to wild-type once drug pressure is removed (Devereux et al. 1999; Verhofstede et al. 1999; Lawrence et al. 2003). For the beneficial trait to become fixed in the population, it must occur in a context where the deleterious trait does not lead to a drop in fitness so severe that it represents an impassable valley in the fitness landscape.

In HIV-1, we are thus observing the viable pathways in the virus' past evolution. These pathways depend on the existence of enabling mutations that increase the protein stability where resistance-associated mutations are later observed. Amino acid replacements can have either stabilizing or destabilizing effects on protein structures, and the magnitude of these changes can be large (Tokuriki et al. 2007). A protein that is only marginally stable can therefore accommodate only a small number of amino acid replacements, since the majority of destabilizing replacements have the potential to result in an unfolded, non-functional protein. Stabilizing replacements therefore have a *general* enabling effect, since they increase the breadth of replacements that can be accommodated at other sites in the protein.

The set of trajectories that lead to drug resistance will be somewhat limited by the constraints arising from protein structure, function, and energetics (Figs 9 and 10) and there are substantial differences in the frequencies by which they lead to drug resistance. Identification of these pathways to resistance has the potential to permit the monitoring of viral sequences with similar properties, that is, variants with a raised probability of contributing to drug resistance. It is important to note that internal nodes on the tree have shared history, and so they are not independent. As a result, the stability effect of some amino acid replacements will be shared by some trajectories. This means that the stability effects in one population are related to and/or influenced by that of others. This non-independence is reflected in Figs 4 and 5 by the shared trajectories, indicated by darker lines. This further emphasises our finding that some evolutionary trajectories are more easily followed than others.

The observed spatial distribution of enabling replacements, spread throughout the protein structure, is further evidence of the general nature of this effect. The alternative method of stabilization, whereby a deleterious intramolecular interaction is relieved by a specific compensating change at a spatially neighbouring site, is rarely observed. Generally stabilizing mutations are therefore enabling rather than directly epistatic. The fact that these patterns are observed for both resistant and non-resistant sequences confirms that the occurrence of enabling mutations may represent a general mechanism of maintaining evolvability (Tokuriki and Tawfik 2009). However, the cumulative energy values of trajectories having drug resistance tend to be higher than those of the non-resistance trajectories. This may be indicative of the fact that the drug resistance conferring change (i.e. adaptive mutations) are selected for because they may be having a fitness/functional advantage thereby compromising stability whereas other changes will not persist if they are very destabilizing to the protein structure (Tokuriki et al. 2008). Also the fact that drug resistance trajectories tend to have more mutations than the non-resistance ones and because these mutations are additive, results in the energy values tending to be higher (Serrano et al. 1993; Zhang et al. 1995).

The compensatory nature of stabilizing mutations has been studied before in viruses (Bloom et al. 2010; Chang and Torbett 2011; Boutwell et al. 2013), and the generality of this evolutionary process is confirmed by the observation of similar trajectories in other systems, such as ribulose-1,5-bisphosphate carboxylase (Gong et al. 2013; Studer et al. 2014), where either neutral or stabilizing replacements were found to facilitate the acquisition of new functions. Similar patterns have also been observed in a number of enzymes from different organisms that acquired new substrate specificities (Tokuriki et al. 2008).

Interestingly, we find no significant differences in the spatial occurrence of amino acid replacements in HIV-1 Pol structures when the pre-HAART sequences are compared with post-HAART sequences. This demonstrates that while there is a difference in the numbers of amino acid replacements (i.e. more changes conferring resistance) before and after drug selection, there is no difference in the pattern of amino acid replacements on the protein structure. We have also shown that these patterns are similar in other HIV-1 enzymes.

In conclusion, the high evolutionary rate of HIV-1 represents a challenge for the development of effective treatments. Since stabilizing mutations in the viral population tend to be enabling, they represent the first steps of a path to drug resistance, especially in patients who are non-compliant with their drug treatment. Moreover, since stabilizing mutations may enable a wide range of subsequent mutations of any type, their occurrence in the viral population should be a cause for concern, since they may allow the virus to evolve in a wide range of unforeseeable directions that are unrelated to drug resistance, such as immune escape. Fortunately, the constraints arising from protein structure (Woo et al. 2010; Snoeck et al. 2011; Williams et al. 2011) limit the ‘choice’ of evolutionary trajectories that the virus can take to acquire a resistance-causing mutation.

## 4. Methods

The HIV-1 drug resistance information was obtained from the Stanford HIV-1 Drug Resistance Database (Shafer 2006), while the HIV-1 subtype B sequences analysed were obtained for the Los Alamos HIV-1 Database (http://www.hiv.lanl.gov/). The search parameters for the amino acid sequence downloads from the Los Alamos HIV Sequence database were: sampling country—US, subtype—B, genomic region—Protease, P51 (RT), and P31 (Integrase). HIV-1 protein structures were obtained from the Protein Data Bank (Berman et al. 2002) with the following data information: protease, PDB code 1KZK (Reiling et al. 2002); reverse transcriptase, PDB code 1RTJ (Esnouf et al. 1995); integrase, PDB code 1ITG (Dyda et al. 1994).

The HIV-1 sequences were split into two sets: (i) prior to 1996, derived before the advent of HAART and (ii) those collected from 1996 onwards, after the widespread use of HAART. After removal of identical sequences, those with internal stop codons and/or undefined amino acids, a total of 649 (prior to 1996) and 4,434 (1996 onwards) sequences were analysed for the protease protein. For the reverse transcriptase, a total of 219 (prior to 1996) and 912 (1996 onwards) sequences were analysed while 105 (prior to 1996) and 737 (1996 onwards) sequences were analysed for the HIV-1 integrase protein. The large size of the 1996-onward data sets (Supplementary Figs S2 and S3) made the ancestral sequence inference computationally intractable, so sequences were split into five sets of 200 randomly selected sequences for both the HIV-1 protease and reverse transcriptase. We maintained the proportion of sequences containing drug resistance mutations in the samples to be consistent with the original data.

The sequences were aligned and the phylogenetic trees were predicted based on maximum likelihood methods implemented in RAxML (Stamatakis 2014), using the WAG substitution model (Whelan and Goldman 2001). Ancestral sequences of HIV-1 proteins were reconstructed using FastML (Ashkenazy et al. 2012), also using the maximum likelihood method and the WAG substitution model. The evolutionary trajectories of every sequence containing at least one drug resistance-associated amino acid replacement were traced to its most recent common ancestor. The sequences of all internal nodes were compared with their ancestors to identify amino acid replacements. The differences between every consecutive pair of nodes were recorded. The phylogenetic tree structure implies that trajectories share subsets of these mutational steps, which can contain single or multiple changes. This information was used to compute the probabilities of acquiring a resistance mutation, given a series of observed changes, by counting the number of trajectories sharing a series of changes that go on to either develop or not develop drug resistance.

The structure of the most recent common ancestor of the sequences for each protein was predicted using Modeller (Sali and Blundell 1993). The change in energy for amino acid replacements was predicted using an empirical force field as implemented in FoldX (Guerois et al. 2002) (version 3 Beta 6). Using the ‘BuildModel’ function in FoldX and with the ‘vdw design’ parameter set to ‘0’ (Schymkowitz et al. 2005), we generated mutant structures for all the sequences occurring in each trajectory, and then predicted the change in energy for each mutant. Because a complete structure for the integrase protein was not available, in this case we conducted a separate analysis using the most complete structure as follows: for every position in which an amino acid replacement was observed, the native amino acid residue in the ‘wild-type’ sequence was mutated to the 19 other possible residues using the BuildModel function in FoldX. The energy change for each observed mutation was also predicted.

The probability of acquisition of a resistance mutation is calculated by dividing the total number of pathways passing through a node that ultimately acquire a resistance mutation by the total number of pathways. For clarity values are omitted if fewer than five sequences pass through a node. The calculation of the probability of acquiring resistance mutation was done as follows: once the ML tree and joint ancestral sequence reconstruction have been performed, the mutational trajectories of each extant sequence can be followed up to the root of the tree. The tree structure invariably means that extant sequences will share some internal nodes. Thus, for each internal node, we can calculate the probability that any sequence having that node on its trajectory will go on to acquire resistance-conferring mutations at some point. Let *N* be the total number of trajectories (resistance-associated or otherwise) passing through a given internal node. Let *N_R_* be the number of these trajectories that acquire resistance at any point downstream of the current node. Then, we estimate the probability *P_R_* of acquiring resistance as:

$$P_{\text{R}}=N_{\text{R}}/N$$

## Supplementary Material

Supplementary Data
