# Supplementary material for: Adaptive HIV-1 evolutionary trajectories are constrained by protein stability
Source: Virus Evol. 2017 Aug 1;3(2):vex019. doi: 10.1093/ve/vex019 (PMC5570062; doi:10.1093/ve/vex019)
Supplement: Supplementary Data [file vex019_supp.pdf]

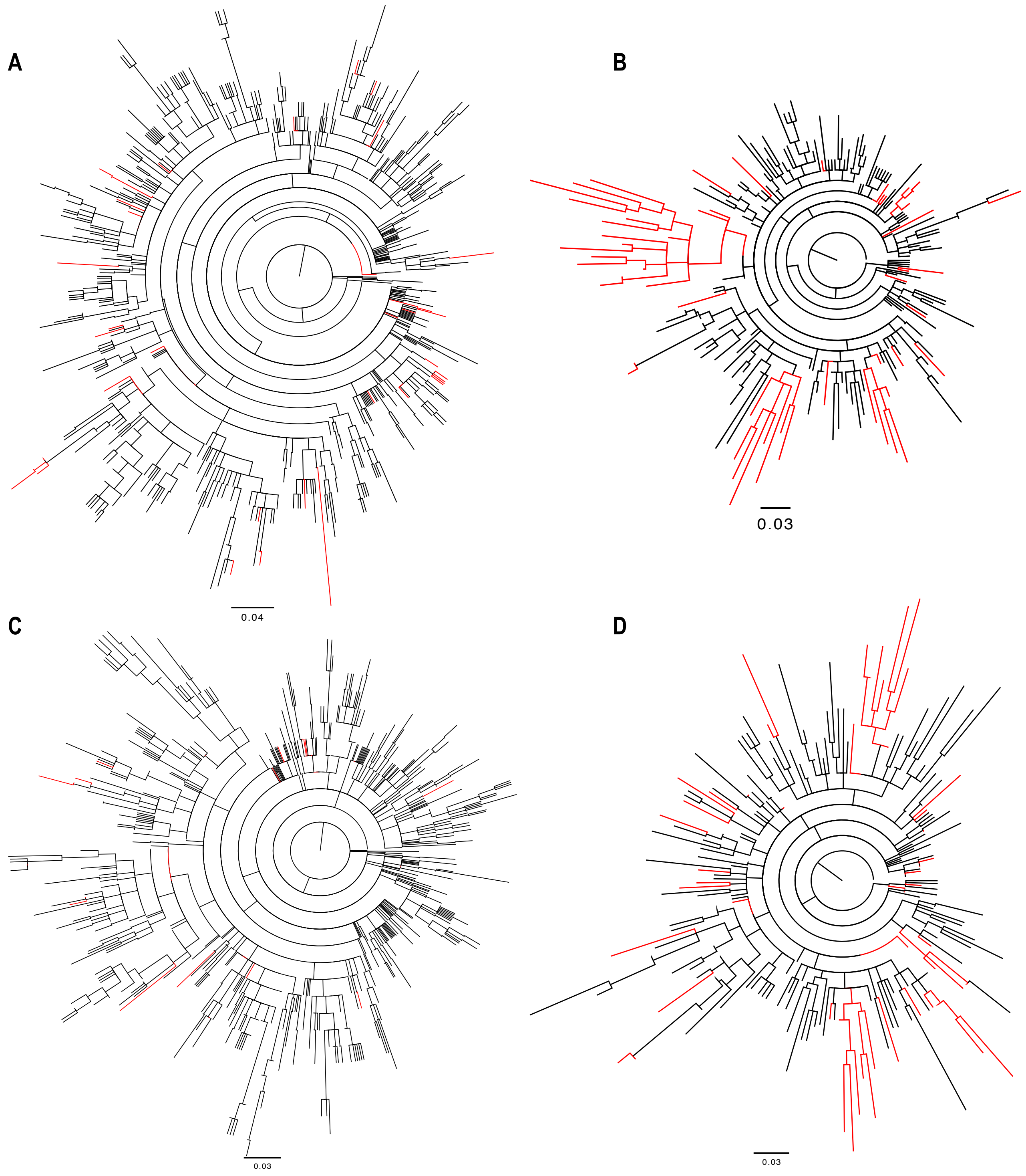

Supplementary Figure 1. Phylogenetic tree showing the evolutionary history of the HIV-1 protease sequences before and after exclusion of drug resistant sites. The pre-HAART/1996 including drug resistance data (A), the post-HAART/1996 onwards sequences including drug resistance data (B), the pre-HAART/1996 excluding drug resistance sites (C) and the post-HAART/1996 onwards sequences excluding drug resistance sites (D). The branches coloured red represents those sequences that have at least one mutation conferring resistance to protease inhibitors, whereas those coloured black have no identified drug resistance mutations. The scale bar indicates the number of amino acid replacements per site.

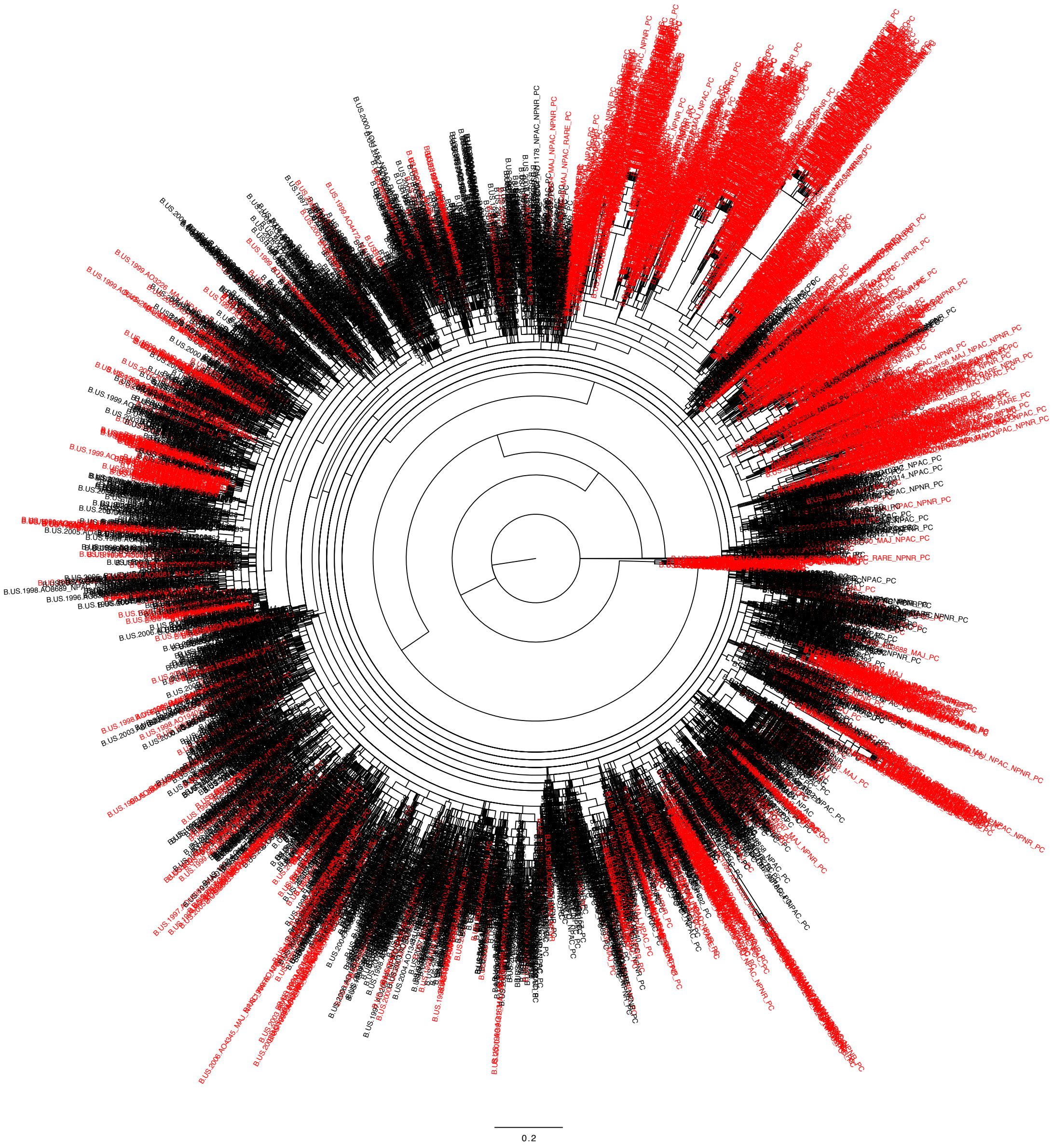

Supplementary Figure 2. Phylogenetic trees showing the evolutionary history of the complete HIV-1 post-HAART/1996 onwards protease subtype B sequences. The branches coloured red represents those sequences that have at least one mutation conferring resistance to protease inhibitors, whereas those coloured black have no identified drug resistance mutations. The scale bar indicates the number of amino acid replacements per site.

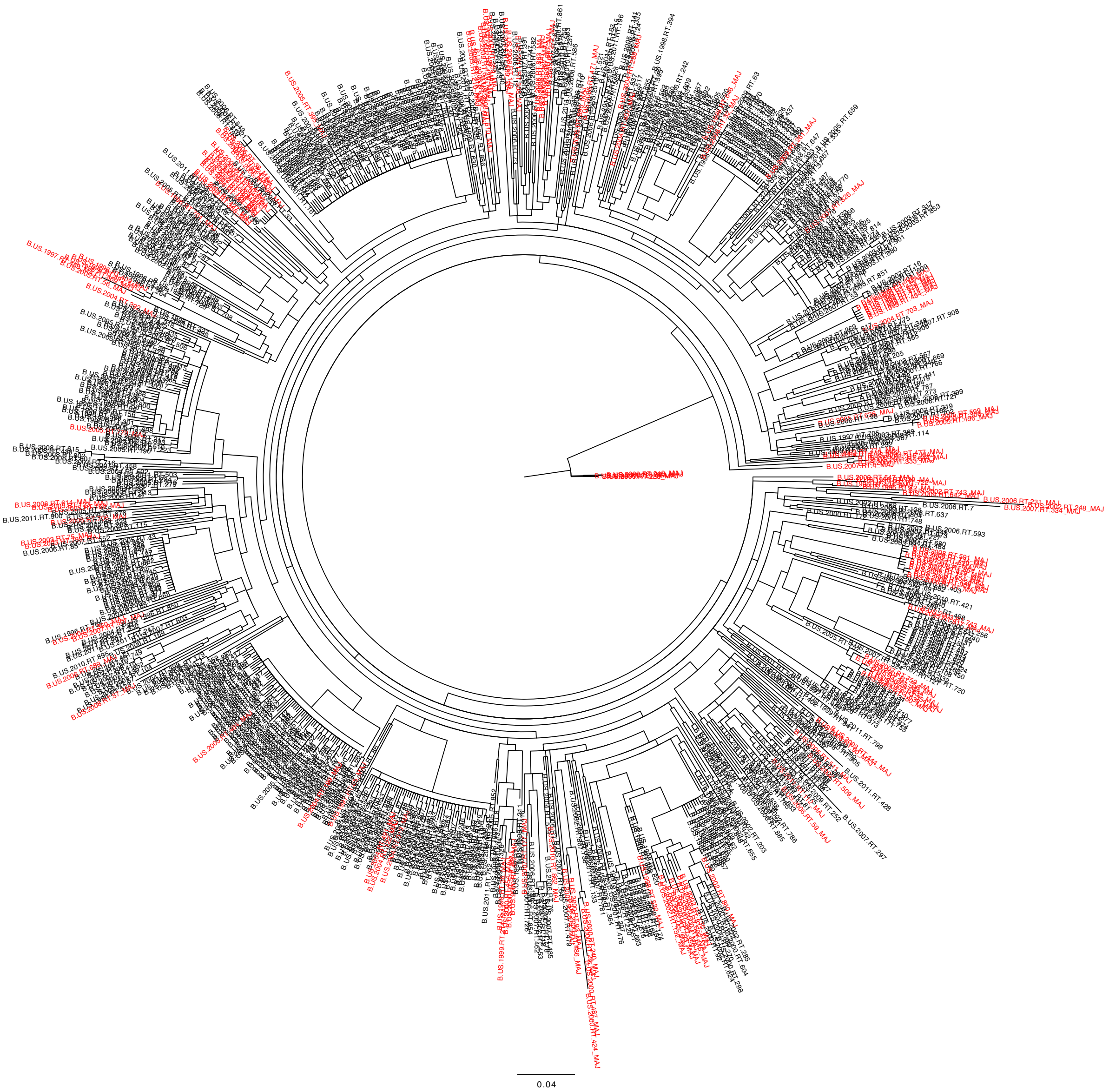

Supplementary Figure 3. Phylogenetic trees showing the evolutionary history of the complete HIV-1 post-HAART/1996 onwards reverse traanscriptase subtype B sequences. The branches coloured red represents those sequences that have at least one mutation conferring resistance to protease inhibitors, whereas those coloured black have no identified drug resistance mutations. The scale bar indicates the number of amino acid replacements per site.

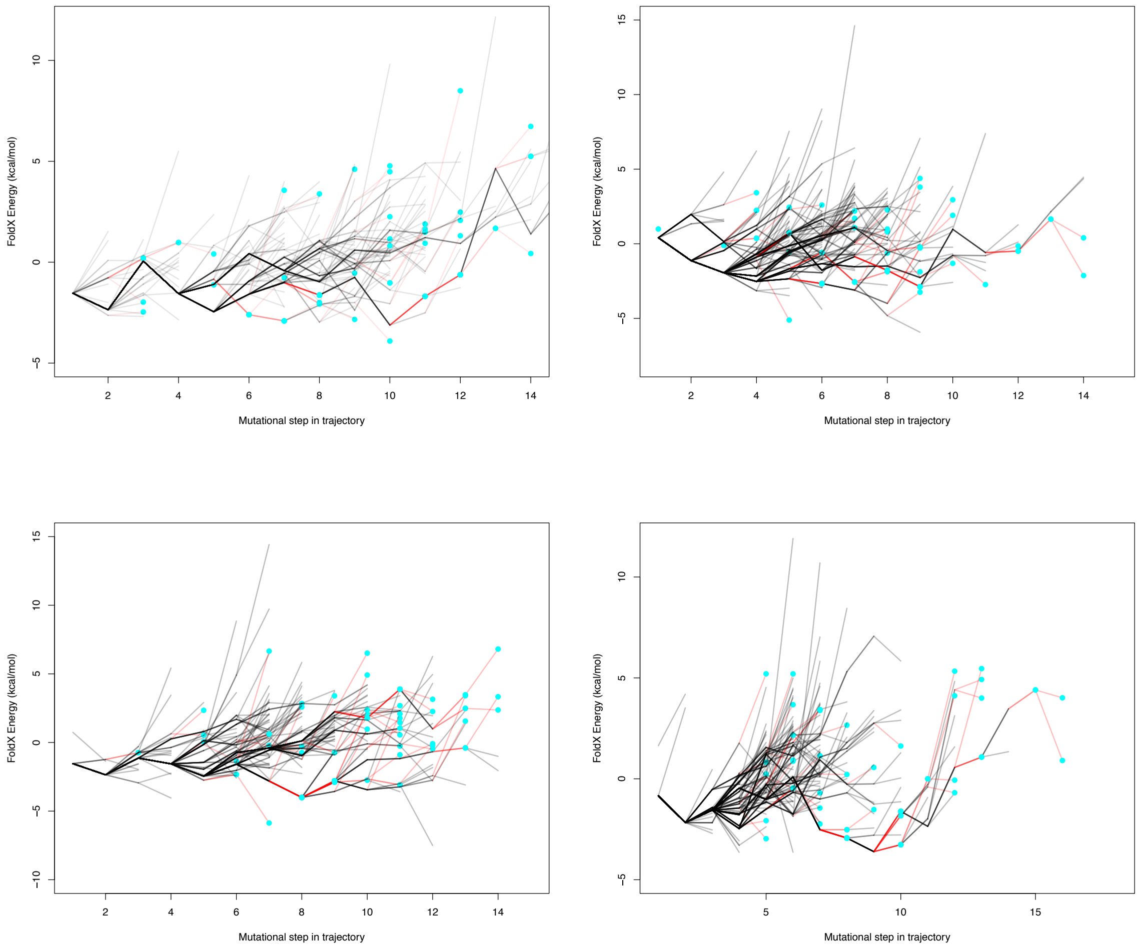

Supplementary Figure 4. Energy landscapes showing trajectory energies for HIV-1 protease protein for both resistant and non-resistant sequences. Each panel represents a data set for one random sample of 200 sequences from the complete data set. Each trajectory is plotted in gray, with frequently shared paths appearing to be darker. The red lines indicate the step in resistance trajectories just before a resistance mutation is acquired (indicated by the blue circles). The y–axis represents the predicted difference in energy between each mutant and the wild type strain (ΔΔG). Each line on the x-axis represents a mutational step between a parent and child node starting from the first descendant of the most recent common ancestor. Note, the energy changes are cumulative.

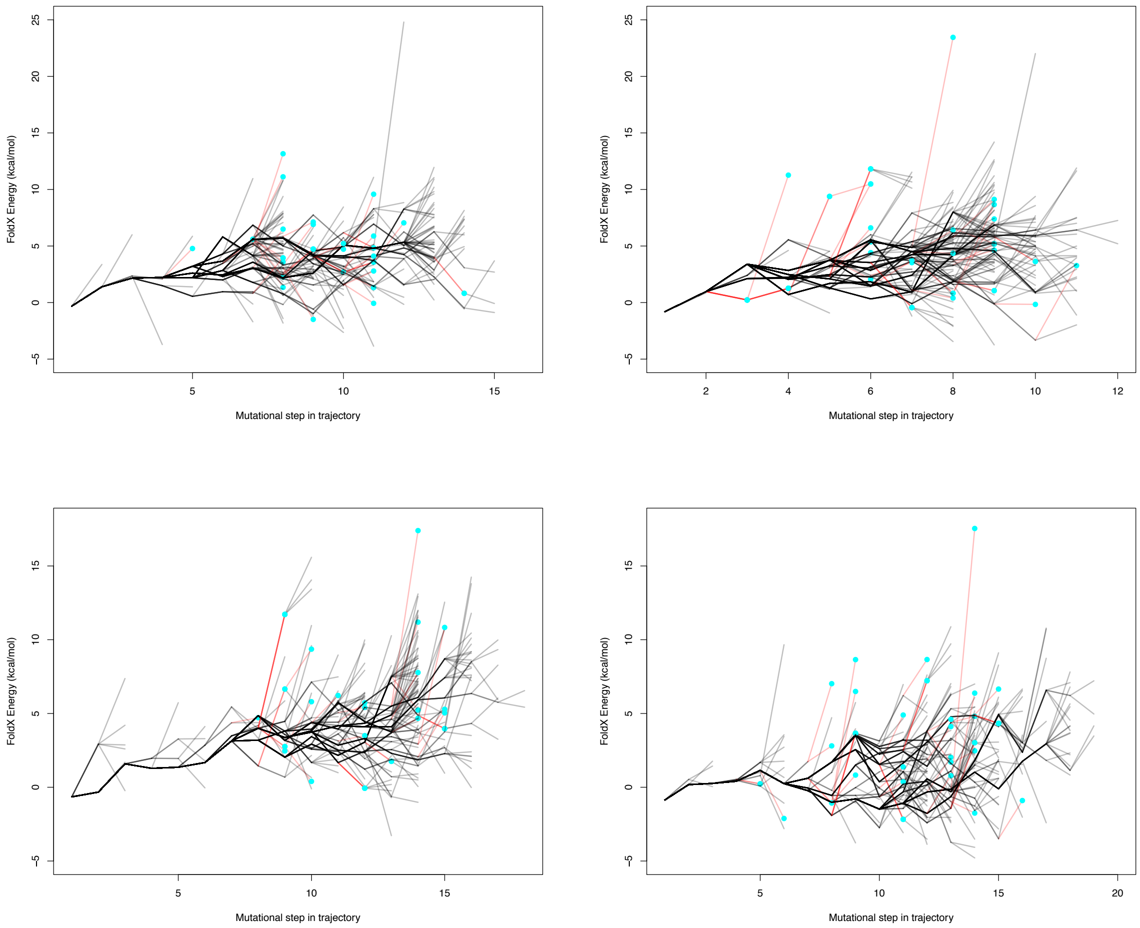

Supplementary Figure 5. Energy landscapes showing trajectory energies for HIV-1 reverse transcriptase protein for both resistant and non-resistant sequences. See supplementary figure 4's legend for details.
